# Tetra­ethyl­ammonium 7,12-di­cyano-1-carba-*closo*-dodeca­borate

**DOI:** 10.1107/S1600536814004759

**Published:** 2014-03-12

**Authors:** Marcus A. Juhasz, Douglas H. Juers, Gregory E. Dwulet, Aaron J. Rosenbaum

**Affiliations:** a Department of Chemistry, Whitman College, Walla Walla, WA 99362, USA; b Department of Physics, Whitman College, Walla Walla, WA 99362, USA

## Abstract

In the title compound, C_8_H_20_N^+^·C_3_H_10_B_11_N_2_^−^, the carborane anion cage displays nearly-perfect *C_s_* symmetry, with the two CN groups lying on a noncrystallographic mirror plane that bis­ects the cage. In the crystal, the anions form extended chains along the *a-*axis direction, with C—H⋯N hydrogen bonds linking consecutive anions. The C N bond lengths (and B—C N angles) in the nitrile moities are 1.1201 (19) Å, 178.60 (15)° and 1.1433 (17) Å, 179.45 (15)°, similar to those observed in organic nitriles. A hydrogen bond between a methylene H atom of the cation and the N atom in one of the nitrile groups of the anion is the closest contact between the anion and cation, at 2.52 Å.

## Related literature   

For the synthesis, and spectroscopic studies of the title compound and the related monosubstituted cyano compound, see: Rosenbaum *et al.* (2013 ▶). For gas phase acidity calculations of cyanated 1-carba-*closo*-dodeca­borate(1−) derivatives, see: Lipping *et al.* (2009 ▶). For studies of 1-carba-*closo*-dodeca­borate(1−) as a weakly coordinating anion, see: Reed (1998 ▶). For the title compound acting as a conjugate base for the strongest Brønsted acids, see: Juhasz *et al.* (2004 ▶). For a general review of the chemistry of the 1-carba-*closo*-dodeca­borate(1−) anion, see: Douvris & Michl (2013 ▶). For bond lengths of cyano groups in organic nitriles, see: Allen *et al.* (1987 ▶).
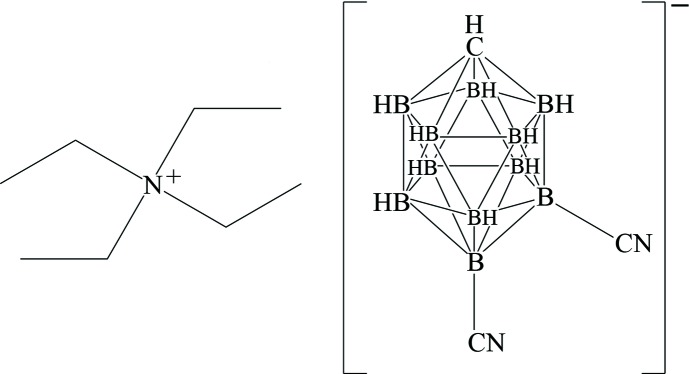


## Experimental   

### 

#### Crystal data  

C_8_H_20_N^+^·C_3_H_10_B_11_N_2_^−^
*M* *_r_* = 323.45
Monoclinic, 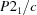

*a* = 8.9280 (2) Å
*b* = 10.5695 (3) Å
*c* = 21.0620 (5) Å
β = 92.165 (2)°
*V* = 1986.09 (8) Å^3^
*Z* = 4
Cu *K*α radiation
μ = 0.40 mm^−1^
*T* = 100 K
0.72 × 0.11 × 0.09 mm

#### Data collection  

Agilent Xcalibur (Onyx, Nova) diffractometer
Absorption correction: multi-scan (*CrysAlis PRO*; Agilent, 2012 ▶) *T*_min_ = 0.874, *T*_max_ = 1.000
13665 measured reflections
3549 independent reflections
3110 reflections with *I* > 2σ(*I*)
*R*_int_ = 0.039

#### Refinement  

*R*[*F* ^2^ > 2σ(*F* ^2^)] = 0.042
*wR*(*F* ^2^) = 0.123
*S* = 1.04
3549 reflections
234 parameters
H atoms treated by a mixture of independent and constrained refinement
Δρ_max_ = 0.20 e Å^−3^
Δρ_min_ = −0.21 e Å^−3^

### 

Data collection: *CrysAlis PRO* (Agilent, 2012 ▶); cell refinement: *CrysAlis PRO*; data reduction: *CrysAlis PRO*; program(s) used to solve structure: *OLEX2* (Dolomanov *et al.*, 2009 ▶); program(s) used to refine structure: *SHELXL97* (Sheldrick, 2008 ▶); molecular graphics: *OLEX2* and *Mercury* (Macrae *et al.*, 2006 ▶); software used to prepare material for publication: *OLEX2* and *publCIF* (Westrip, 2010 ▶).

## Supplementary Material

Crystal structure: contains datablock(s) I. DOI: 10.1107/S1600536814004759/lr2122sup1.cif

Structure factors: contains datablock(s) I. DOI: 10.1107/S1600536814004759/lr2122Isup2.hkl

Supporting information file. DOI: 10.1107/S1600536814004759/lr2122Isup3.cdx

CCDC reference: 989485

Additional supporting information:  crystallographic information; 3D view; checkCIF report

## Figures and Tables

**Table 1:** Hydrogen-bond geometry (Å, °)

*D*—H⋯*A*	*D*—H	H⋯*A*	*D*⋯*A*	*D*—H⋯*A*
C1—H1⋯N16^i^	0.968 (18)	2.496 (18)	3.3042 (18)	140.9 (14)
C18—H18a⋯N14	0.97	2.52	3.4323 (17)	157

Symmetry code: (i) 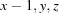.

**Fig. 1.**
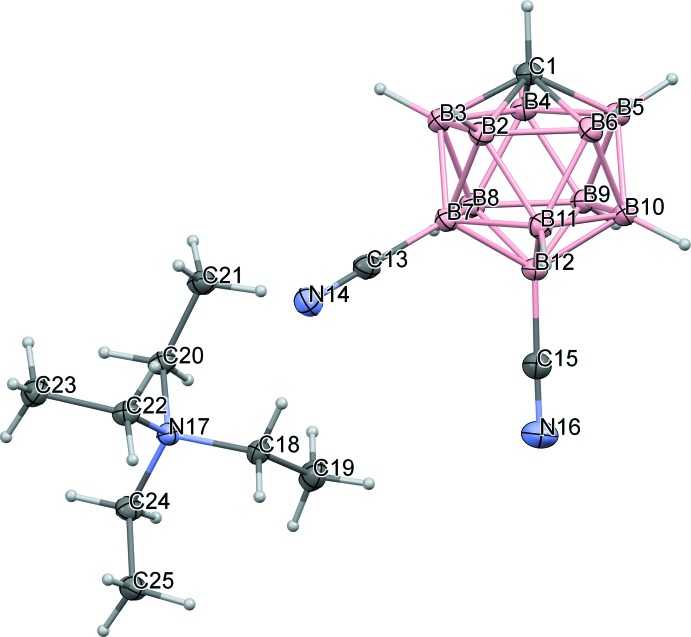
Structure of the repeat unit of tetraethylammonium 7,12-dicyano-1-carba-closo-dodecaborate. Thermal ellipsoids are at the 40% probability level.

**Fig. 2.**
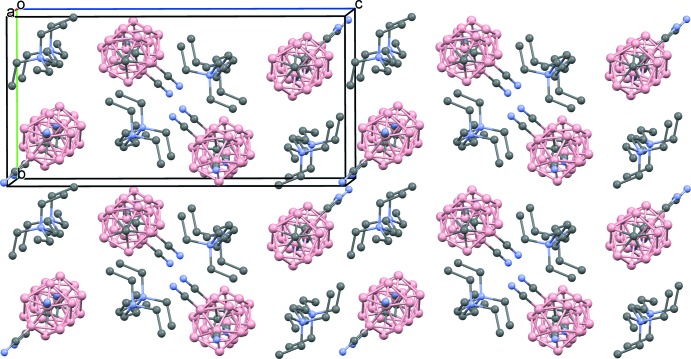
Packing diagram for tetraethylammonium 7,12-dicyano-1-carba-closo-dodecaborate.

**Fig. 3.**
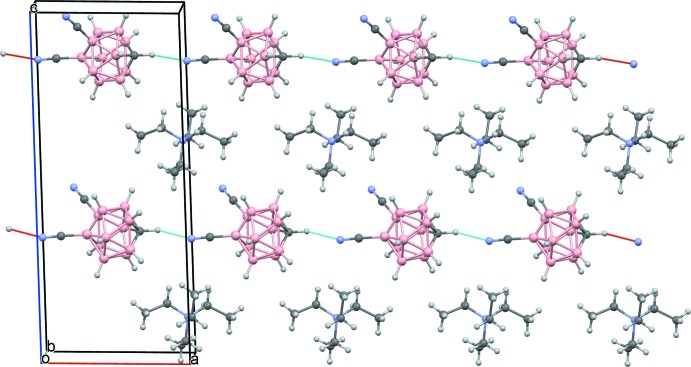
Partial view along the b axis of the crystal, showing the hydrogen-bonded chains of carborane anions.

## supplementary crystallographic information

### 1. Comment

Derivatives of the 1-carba-*closo*-dodecaborate(1-) carborane anion, e.g. CB_11_H_12_^-^, have been recognized as exceptionally weakly-coordinating anions (Reed, 1998) and they have been used to prepare the strongest Brønsted acids known (Juhasz *et al.*, 2004). This family of carborane anions has further potential uses in pharmaceuticals, in optical and electronic materials, and in catalysts for industrial-scale chemical reactions. A relatively small number of synthetic reactions have been developed for producing new derivatives of CB_11_H_12_^-^, and derivatives bearing CN groups on boron were unknown until very recently (Rosenbaum *et al.*, 2013). In the present report, we describe the crystal structure of the tetra­ethyl­ammonium salt of the di­cyanated carborane anion, 7,12-(CN)_2_-*closo*-CHB_11_H_9_^-^. In the crystal structure, the carborane anion cluster has nearly perfect *C_s_* symmetry, with the two CN groups lying on a mirror plane that bis­ects the cluster. The carborane anions pack to form extended chains. The closest contact between consecutive anions is hydrogen bond with a length of 2.406 Å from hydrogen on C1 of one cluster to nitro­gen in the CN group on B12 of the next. A weak inter­action between the anion and cation is indicated; the closest contact between these involves a methyl­ene hydrogen on the tetra­ethyl­ammonium cation at 2.519 Å from the nitro­gen atom in the CN group on B7 of the carborane anion. The C≡N bond distances (and B—C≡N angles) are 1.1201 (19) Å, 178.60 (15)° and 1.1433 (17) Å, 179.45 (15)° for the CN groups on B12 and B7, respectively. These bond lengths are similar to those observed in organic nitriles (Allen *et al.*, 1987).

### 2. Experimental

For the synthesis and spectroscopic characterization of the title compound, see (Rosenbaum *et al.*, 2013). Colorless crystals of the compound suitable for X-ray diffraction were obtained by the slow evaporation over 10 days of a saturated solution of the compound in a 1:3 aceto­nitrile/water solution. The crystallization procedure was performed under normal atmosphere at 25 °C using reagent grade aceto­nitrile and deionized water.

### 2.1. Refinement

Crystal data, data collection and structure refinement details are summarized in Table 1. All H-atoms were positioned and refined using a riding model with d(B—H)= 1.10 Å, *U*_iso_ = 1.2*U*_eq_(B) for B—H bonds; d(C—H)= 0.97 Å, *U*_iso_ = 1.2*U*_eq_(C) for C—H and CH_2_ groups and d(C—H)= 0.96 Å, *U*_iso_ = 1.5*U*_eq_(C) for CH_3_ group; except for the hydrogen atom of the C1 carbon atom which was refined isotropically.

### Crystal data

**Table:**

C_8_H_20_N^+^·C_3_H_10_B_11_N_2_^−^	*F*(000) = 688
*M**_r_* = 323.45	*D*_x_ = 1.081 Mg m^−^^3^
Monoclinic, *P*2_1_/*c*	Cu *K*α radiation, λ = 1.5418 Å
*a* = 8.9280 (2) Å	Cell parameters from 9624 reflections
*b* = 10.5695 (3) Å	θ = 4.2–67.0°
*c* = 21.0620 (5) Å	µ = 0.40 mm^−^^1^
β = 92.165 (2)°	*T* = 100 K
*V* = 1986.09 (8) Å^3^	Needle, clear light colourless
*Z* = 4	0.72 × 0.11 × 0.09 mm

### Data collection

**Table:**

Agilent Xcalibur (Onyx, Nova) diffractometer	3549 independent reflections
Radiation source: Nova (Cu) X-ray Source	3110 reflections with *I* > 2σ(*I*)
Mirror monochromator	*R*_int_ = 0.039
Detector resolution: 8.3552 pixels mm^-1^	θ_max_ = 67.1°, θ_min_ = 4.2°
ω scans	*h* = −10→10
Absorption correction: multi-scan (*CrysAlis PRO*; Agilent, 2012)	*k* = −12→12
*T*_min_ = 0.874, *T*_max_ = 1.000	*l* = −25→18
13665 measured reflections	

### Refinement

**Table:**

Refinement on *F*^2^	0 restraints
Least-squares matrix: full	Primary atom site location: iterative
*R*[*F*^2^ > 2σ(*F*^2^)] = 0.042	H atoms treated by a mixture of independent and constrained refinement
*wR*(*F*^2^) = 0.123	*w* = 1/[σ^2^(*F*_o_^2^) + (0.0746*P*)^2^ + 0.5426*P*] where *P* = (*F*_o_^2^ + 2*F*_c_^2^)/3
*S* = 1.04	(Δ/σ)_max_ = 0.001
3549 reflections	Δρ_max_ = 0.20 e Å^−^^3^
234 parameters	Δρ_min_ = −0.21 e Å^−^^3^

### Special details

**Table:**

Experimental. Absorption correction: CrysAlisPro, Agilent Technologies, Empirical absorption correction using spherical harmonics, implemented in SCALE3 ABSPACK scaling algorithm.
Geometry. All e.s.d.'s (except the e.s.d. in the dihedral angle between two l.s. planes) are estimated using the full covariance matrix. The cell e.s.d.'s are taken into account individually in the estimation of e.s.d.'s in distances, angles and torsion angles; correlations between e.s.d.'s in cell parameters are only used when they are defined by crystal symmetry. An approximate (isotropic) treatment of cell e.s.d.'s is used for estimating e.s.d.'s involving l.s. planes.

### Fractional atomic coordinates and isotropic or equivalent isotropic displacement parameters (Å^2^)

**Table:**

	*x*	*y*	*z*	*U*_iso_*/*U*_eq_	
N17	1.01231 (11)	0.69866 (9)	0.37094 (5)	0.0192 (2)	
N14	0.76135 (13)	0.55607 (11)	0.51262 (5)	0.0293 (3)	
C13	0.69013 (14)	0.61804 (12)	0.54418 (6)	0.0232 (3)	
C1	0.32003 (14)	0.76849 (12)	0.62912 (6)	0.0226 (3)	
H1	0.213 (2)	0.7535 (17)	0.6265 (9)	0.044 (5)*	
C20	0.88278 (13)	0.77399 (12)	0.34123 (6)	0.0234 (3)	
H20A	0.9091	0.8630	0.3426	0.028*	
H20B	0.8704	0.7500	0.2969	0.028*	
C18	1.03464 (14)	0.72809 (12)	0.44147 (6)	0.0231 (3)	
H18A	0.9447	0.7038	0.4628	0.028*	
H18B	1.1163	0.6764	0.4587	0.028*	
C22	0.98194 (14)	0.55670 (11)	0.36737 (6)	0.0218 (3)	
H22A	1.0667	0.5128	0.3873	0.026*	
H22B	0.8950	0.5383	0.3920	0.026*	
C25	1.29534 (14)	0.67411 (13)	0.35946 (6)	0.0255 (3)	
H25A	1.3179	0.7028	0.4020	0.038*	
H25B	1.3752	0.6977	0.3326	0.038*	
H25C	1.2847	0.5837	0.3594	0.038*	
C24	1.15065 (13)	0.73406 (12)	0.33489 (6)	0.0227 (3)	
H24A	1.1340	0.7102	0.2907	0.027*	
H24B	1.1623	0.8253	0.3363	0.027*	
C23	0.95487 (15)	0.50319 (13)	0.30122 (7)	0.0303 (3)	
H23A	0.8654	0.5397	0.2823	0.045*	
H23B	0.9434	0.4130	0.3037	0.045*	
H23C	1.0386	0.5229	0.2757	0.045*	
C21	0.73378 (14)	0.75717 (13)	0.37238 (7)	0.0270 (3)	
H21A	0.7071	0.6692	0.3722	0.041*	
H21B	0.6579	0.8046	0.3493	0.041*	
H21C	0.7420	0.7870	0.4154	0.041*	
C15	0.84647 (15)	0.84551 (13)	0.64203 (6)	0.0276 (3)	
N16	0.97050 (14)	0.86121 (14)	0.64645 (7)	0.0423 (3)	
B11	0.59691 (15)	0.68338 (13)	0.67214 (6)	0.0214 (3)	
H11	0.6671	0.6123	0.6971	0.026*	
C19	1.06859 (17)	0.86511 (14)	0.45676 (7)	0.0336 (3)	
H19A	1.1611	0.8889	0.4383	0.050*	
H19B	1.0772	0.8759	0.5020	0.050*	
H19C	0.9890	0.9175	0.4397	0.050*	
B3	0.41460 (15)	0.75312 (14)	0.56090 (6)	0.0222 (3)	
H3	0.3644	0.7261	0.5144	0.027*	
B5	0.38243 (15)	0.88617 (14)	0.67872 (7)	0.0226 (3)	
H5	0.3109	0.9451	0.7081	0.027*	
B2	0.43587 (16)	0.64087 (14)	0.62446 (7)	0.0223 (3)	
H2	0.3995	0.5419	0.6187	0.027*	
B4	0.38189 (15)	0.90407 (14)	0.59491 (7)	0.0225 (3)	
H4	0.3100	0.9745	0.5703	0.027*	
B8	0.56256 (15)	0.86592 (13)	0.56845 (7)	0.0216 (3)	
H8	0.6105	0.9117	0.5269	0.026*	
B10	0.56283 (15)	0.83638 (14)	0.70569 (6)	0.0218 (3)	
H10	0.6108	0.8638	0.7526	0.026*	
B9	0.54205 (15)	0.94840 (13)	0.64211 (7)	0.0217 (3)	
H9	0.5768	1.0477	0.6479	0.026*	
B6	0.41595 (16)	0.72351 (14)	0.69686 (7)	0.0233 (3)	
H6	0.3661	0.6776	0.7379	0.028*	
B7	0.59456 (15)	0.70269 (13)	0.58752 (6)	0.0204 (3)	
B12	0.67289 (15)	0.82232 (13)	0.63756 (6)	0.0205 (3)	

### Atomic displacement parameters (Å^2^)

**Table:**

	*U*^11^	*U*^22^	*U*^33^	*U*^12^	*U*^13^	*U*^23^
N17	0.0172 (5)	0.0209 (5)	0.0194 (5)	−0.0007 (4)	−0.0004 (4)	0.0010 (4)
N14	0.0295 (6)	0.0313 (6)	0.0273 (6)	0.0027 (5)	0.0039 (5)	0.0012 (5)
C13	0.0208 (6)	0.0265 (6)	0.0222 (6)	0.0004 (5)	0.0000 (5)	0.0045 (5)
C1	0.0163 (6)	0.0268 (6)	0.0246 (6)	−0.0010 (5)	0.0004 (5)	−0.0015 (5)
C20	0.0203 (6)	0.0233 (6)	0.0262 (6)	0.0016 (5)	−0.0039 (5)	0.0021 (5)
C18	0.0215 (6)	0.0292 (7)	0.0184 (6)	0.0014 (5)	−0.0010 (5)	−0.0019 (5)
C22	0.0201 (6)	0.0190 (6)	0.0262 (6)	−0.0008 (5)	0.0000 (5)	0.0011 (5)
C25	0.0209 (6)	0.0310 (7)	0.0248 (6)	0.0014 (5)	0.0039 (5)	0.0025 (5)
C24	0.0211 (6)	0.0248 (6)	0.0224 (6)	−0.0030 (5)	0.0028 (5)	0.0038 (5)
C23	0.0316 (7)	0.0279 (7)	0.0309 (7)	−0.0007 (6)	−0.0042 (5)	−0.0054 (5)
C21	0.0198 (6)	0.0277 (7)	0.0333 (7)	0.0025 (5)	−0.0025 (5)	−0.0011 (5)
C15	0.0244 (7)	0.0306 (7)	0.0279 (7)	0.0012 (5)	0.0005 (5)	−0.0014 (5)
N16	0.0238 (7)	0.0541 (9)	0.0492 (8)	0.0000 (6)	0.0020 (5)	−0.0046 (6)
B11	0.0219 (7)	0.0233 (7)	0.0189 (6)	0.0017 (5)	0.0010 (5)	0.0013 (5)
C19	0.0356 (7)	0.0302 (7)	0.0346 (8)	0.0052 (6)	−0.0069 (6)	−0.0099 (6)
B3	0.0195 (7)	0.0269 (7)	0.0200 (7)	0.0006 (5)	−0.0009 (5)	−0.0004 (5)
B5	0.0183 (6)	0.0263 (7)	0.0233 (7)	0.0003 (5)	0.0014 (5)	−0.0016 (6)
B2	0.0215 (7)	0.0235 (7)	0.0219 (7)	−0.0017 (5)	0.0020 (5)	−0.0002 (5)
B4	0.0188 (6)	0.0242 (7)	0.0244 (7)	0.0010 (5)	−0.0010 (5)	0.0008 (6)
B8	0.0192 (6)	0.0241 (7)	0.0216 (7)	0.0005 (5)	0.0004 (5)	0.0028 (5)
B10	0.0189 (6)	0.0264 (7)	0.0199 (6)	0.0014 (5)	−0.0006 (5)	−0.0014 (5)
B9	0.0177 (6)	0.0226 (7)	0.0246 (7)	0.0000 (5)	0.0000 (5)	−0.0004 (5)
B6	0.0226 (7)	0.0266 (7)	0.0209 (7)	−0.0011 (6)	0.0034 (5)	0.0008 (5)
B7	0.0195 (6)	0.0228 (7)	0.0189 (6)	0.0008 (5)	0.0013 (5)	0.0008 (5)
B12	0.0158 (6)	0.0231 (7)	0.0226 (7)	−0.0001 (5)	0.0004 (5)	−0.0003 (5)

### Geometric parameters (Å, º)

**Table:**

N17—C20	1.5192 (15)	B11—B2	1.780 (2)
N17—C24	1.5208 (15)	B11—B12	1.785 (2)
N17—C18	1.5234 (15)	B11—B7	1.7932 (19)
N17—C22	1.5261 (15)	B11—B10	1.795 (2)
N14—C13	1.1433 (17)	B11—H11	1.1000
C13—B7	1.5558 (18)	C19—H19A	0.9600
C1—B3	1.7016 (18)	C19—H19B	0.9600
C1—B6	1.7037 (19)	C19—H19C	0.9600
C1—B5	1.7043 (18)	B3—B7	1.7640 (19)
C1—B2	1.7048 (19)	B3—B4	1.778 (2)
C1—B4	1.7052 (19)	B3—B8	1.7821 (19)
C1—H1	0.965 (19)	B3—B2	1.793 (2)
C20—C21	1.5158 (18)	B3—H3	1.1000
C20—H20A	0.9700	B5—B10	1.7676 (19)
C20—H20B	0.9700	B5—B9	1.7723 (19)
C18—C19	1.5117 (19)	B5—B4	1.7751 (19)
C18—H18A	0.9700	B5—B6	1.784 (2)
C18—H18B	0.9700	B5—H5	1.1000
C22—C23	1.5146 (18)	B2—B7	1.7672 (19)
C22—H22A	0.9700	B2—B6	1.7721 (19)
C22—H22B	0.9700	B2—H2	1.1000
C25—C24	1.5125 (17)	B4—B8	1.7728 (19)
C25—H25A	0.9600	B4—B9	1.7734 (19)
C25—H25B	0.9600	B4—H4	1.1000
C25—H25C	0.9600	B8—B12	1.7869 (19)
C24—H24A	0.9700	B8—B7	1.7919 (19)
C24—H24B	0.9700	B8—B9	1.7950 (19)
C23—H23A	0.9600	B8—H8	1.1000
C23—H23B	0.9600	B10—B12	1.7757 (18)
C23—H23C	0.9600	B10—B6	1.777 (2)
C21—H21A	0.9600	B10—B9	1.7918 (19)
C21—H21B	0.9600	B10—H10	1.1000
C21—H21C	0.9600	B9—B12	1.7771 (19)
C15—N16	1.1201 (19)	B9—H9	1.1000
C15—B12	1.5683 (18)	B6—H6	1.1000
B11—B6	1.7677 (19)	B7—B12	1.7727 (19)
			
C20—N17—C24	106.58 (9)	B7—B2—B6	107.74 (10)
C20—N17—C18	111.36 (9)	C1—B2—B11	104.28 (10)
C24—N17—C18	110.96 (9)	B7—B2—B11	60.73 (8)
C20—N17—C22	111.34 (9)	B6—B2—B11	59.69 (8)
C24—N17—C22	111.31 (9)	C1—B2—B3	58.14 (8)
C18—N17—C22	105.38 (9)	B7—B2—B3	59.39 (8)
N14—C13—B7	179.45 (15)	B6—B2—B3	107.75 (10)
B3—C1—B6	115.51 (10)	B11—B2—B3	108.34 (10)
B3—C1—B5	115.22 (10)	C1—B2—H2	125.5
B6—C1—B5	63.13 (8)	B7—B2—H2	122.8
B3—C1—B2	63.54 (8)	B6—B2—H2	121.6
B6—C1—B2	62.65 (8)	B11—B2—H2	122.0
B5—C1—B2	115.33 (10)	B3—B2—H2	121.6
B3—C1—B4	62.90 (8)	C1—B4—B8	104.76 (10)
B6—C1—B4	115.32 (10)	C1—B4—B9	104.62 (10)
B5—C1—B4	62.75 (8)	B8—B4—B9	60.82 (8)
B2—C1—B4	115.70 (10)	C1—B4—B5	58.60 (8)
B3—C1—H1	117.1 (11)	B8—B4—B5	108.67 (10)
B6—C1—H1	117.8 (11)	B9—B4—B5	59.93 (8)
B5—C1—H1	117.0 (11)	C1—B4—B3	58.45 (8)
B2—C1—H1	117.8 (11)	B8—B4—B3	60.26 (8)
B4—C1—H1	116.7 (11)	B9—B4—B3	108.77 (10)
C21—C20—N17	115.28 (10)	B5—B4—B3	108.10 (10)
C21—C20—H20A	108.5	C1—B4—H4	125.1
N17—C20—H20A	108.5	B8—B4—H4	121.8
C21—C20—H20B	108.5	B9—B4—H4	122.0
N17—C20—H20B	108.5	B5—B4—H4	121.4
H20A—C20—H20B	107.5	B3—B4—H4	121.3
C19—C18—N17	114.90 (11)	B4—B8—B3	60.00 (8)
C19—C18—H18A	108.5	B4—B8—B12	106.42 (9)
N17—C18—H18A	108.5	B3—B8—B12	106.50 (10)
C19—C18—H18B	108.5	B4—B8—B7	106.72 (10)
N17—C18—H18B	108.5	B3—B8—B7	59.15 (8)
H18A—C18—H18B	107.5	B12—B8—B7	59.38 (8)
C23—C22—N17	115.74 (10)	B4—B8—B9	59.61 (8)
C23—C22—H22A	108.3	B3—B8—B9	107.61 (9)
N17—C22—H22A	108.3	B12—B8—B9	59.49 (8)
C23—C22—H22B	108.3	B7—B8—B9	107.12 (9)
N17—C22—H22B	108.3	B4—B8—H8	122.4
H22A—C22—H22B	107.4	B3—B8—H8	122.2
C24—C25—H25A	109.5	B12—B8—H8	122.8
C24—C25—H25B	109.5	B7—B8—H8	122.5
H25A—C25—H25B	109.5	B9—B8—H8	121.9
C24—C25—H25C	109.5	B5—B10—B12	106.89 (9)
H25A—C25—H25C	109.5	B5—B10—B6	60.43 (8)
H25B—C25—H25C	109.5	B12—B10—B6	106.73 (10)
C25—C24—N17	115.18 (10)	B5—B10—B9	59.72 (8)
C25—C24—H24A	108.5	B12—B10—B9	59.75 (8)
N17—C24—H24A	108.5	B6—B10—B9	108.07 (9)
C25—C24—H24B	108.5	B5—B10—B11	107.92 (10)
N17—C24—H24B	108.5	B12—B10—B11	59.96 (8)
H24A—C24—H24B	107.5	B6—B10—B11	59.31 (8)
C22—C23—H23A	109.5	B9—B10—B11	108.38 (9)
C22—C23—H23B	109.5	B5—B10—H10	121.9
H23A—C23—H23B	109.5	B12—B10—H10	122.6
C22—C23—H23C	109.5	B6—B10—H10	122.1
H23A—C23—H23C	109.5	B9—B10—H10	121.5
H23B—C23—H23C	109.5	B11—B10—H10	121.6
C20—C21—H21A	109.5	B5—B9—B4	60.08 (8)
C20—C21—H21B	109.5	B5—B9—B12	106.63 (10)
H21A—C21—H21B	109.5	B4—B9—B12	106.82 (10)
C20—C21—H21C	109.5	B5—B9—B10	59.46 (8)
H21A—C21—H21C	109.5	B4—B9—B10	107.63 (10)
H21B—C21—H21C	109.5	B12—B9—B10	59.68 (7)
N16—C15—B12	178.60 (15)	B5—B9—B8	107.81 (10)
B6—B11—B2	59.93 (8)	B4—B9—B8	59.58 (8)
B6—B11—B12	106.76 (10)	B12—B9—B8	60.03 (8)
B2—B11—B12	106.79 (9)	B10—B9—B8	108.30 (10)
B6—B11—B7	106.79 (10)	B5—B9—H9	122.2
B2—B11—B7	59.28 (7)	B4—B9—H9	122.2
B12—B11—B7	59.40 (8)	B12—B9—H9	122.6
B6—B11—B10	59.84 (8)	B10—B9—H9	121.7
B2—B11—B10	107.70 (10)	B8—B9—H9	121.5
B12—B11—B10	59.47 (8)	C1—B6—B11	104.86 (9)
B7—B11—B10	107.02 (10)	C1—B6—B2	58.71 (8)
B6—B11—H11	122.3	B11—B6—B2	60.38 (8)
B2—B11—H11	122.1	C1—B6—B10	104.23 (10)
B12—B11—H11	122.6	B11—B6—B10	60.85 (8)
B7—B11—H11	122.6	B2—B6—B10	108.86 (10)
B10—B11—H11	121.9	C1—B6—B5	58.45 (8)
C18—C19—H19A	109.5	B11—B6—B5	108.42 (10)
C18—C19—H19B	109.5	B2—B6—B5	108.20 (10)
H19A—C19—H19B	109.5	B10—B6—B5	59.52 (8)
C18—C19—H19C	109.5	C1—B6—H6	125.2
H19A—C19—H19C	109.5	B11—B6—H6	121.8
H19B—C19—H19C	109.5	B2—B6—H6	121.0
C1—B3—B7	103.62 (9)	B10—B6—H6	122.2
C1—B3—B4	58.65 (8)	B5—B6—H6	121.6
B7—B3—B4	107.74 (10)	C13—B7—B3	120.02 (10)
C1—B3—B8	104.51 (10)	C13—B7—B2	120.81 (11)
B7—B3—B8	60.70 (8)	B3—B7—B2	61.05 (8)
B4—B3—B8	59.74 (8)	C13—B7—B12	123.11 (11)
C1—B3—B2	58.32 (8)	B3—B7—B12	107.91 (10)
B7—B3—B2	59.56 (8)	B2—B7—B12	107.87 (10)
B4—B3—B2	107.90 (10)	C13—B7—B8	120.56 (10)
B8—B3—B2	108.59 (9)	B3—B7—B8	60.15 (8)
C1—B3—H3	125.4	B2—B7—B8	109.33 (10)
B7—B3—H3	122.7	B12—B7—B8	60.17 (8)
B4—B3—H3	121.6	C13—B7—B11	122.11 (11)
B8—B3—H3	121.9	B3—B7—B11	109.06 (9)
B2—B3—H3	121.5	B2—B7—B11	59.99 (8)
C1—B5—B10	104.62 (10)	B12—B7—B11	60.05 (8)
C1—B5—B9	104.70 (9)	B8—B7—B11	109.16 (10)
B10—B5—B9	60.82 (8)	C15—B12—B7	120.93 (11)
C1—B5—B4	58.65 (8)	C15—B12—B10	120.93 (11)
B10—B5—B4	108.63 (10)	B7—B12—B10	108.80 (10)
B9—B5—B4	59.99 (8)	C15—B12—B9	121.98 (11)
C1—B5—B6	58.42 (8)	B7—B12—B9	108.76 (9)
B10—B5—B6	60.06 (8)	B10—B12—B9	60.57 (8)
B9—B5—B6	108.64 (10)	C15—B12—B11	119.61 (11)
B4—B5—B6	108.05 (10)	B7—B12—B11	60.54 (8)
C1—B5—H5	125.1	B10—B12—B11	60.57 (8)
B10—B5—H5	121.9	B9—B12—B11	109.53 (9)
B9—B5—H5	121.9	C15—B12—B8	121.44 (11)
B4—B5—H5	121.3	B7—B12—B8	60.45 (8)
B6—B5—H5	121.4	B10—B12—B8	109.38 (9)
C1—B2—B7	103.35 (10)	B9—B12—B8	60.48 (8)
C1—B2—B6	58.64 (8)	B11—B12—B8	109.78 (10)

### Hydrogen-bond geometry (Å, º)

**Table:**

*D*—H···*A*	*D*—H	H···*A*	*D*···*A*	*D*—H···*A*
C1—H1···N16^i^	0.968 (18)	2.496 (18)	3.3042 (18)	140.9 (14)
C18—H18a···N14	0.97	2.52	3.4323 (17)	157

Symmetry code: (i) *x*−1, *y*, *z*.
